# Predicting kinetics of water-rich permeate flux through photocatalytic mesh under visible light illumination

**DOI:** 10.1038/s41598-021-00607-w

**Published:** 2021-10-26

**Authors:** Bishwash Shrestha, Mohammadamin Ezazi, Seyed Vahid Rad, Gibum Kwon

**Affiliations:** Department of Mechanical Engineering, University of Kansas, Lawrence, KS 66045 USA

**Keywords:** Photocatalysis, Pollution remediation, Nanoparticles

## Abstract

Membrane-based separation technologies are attractive to remediating unconventional water sources, including brackish, industrial, and municipal wastewater, due to their versatility and relatively high energy efficiency. However, membrane fouling by dissolved or suspended organic substances remains a primary challenge which can result in an irreversible decline of the permeate flux. To overcome this, membranes have been incorporated with photocatalytic materials that can degrade these organic substances deposited on the surface upon light illumination. While such photocatalytic membranes have demonstrated that they can recover their inherent permeability, less information is known about the effect of photocatalysis on the kinetics of the permeate flux. In this work, a photocatalytic mesh that can selectively permeate water while repelling oil was fabricated by coating a mixture of nitrogen-doped TiO_2_ (N-TiO_2_) and perfluorosilane-grafted SiO_2_ (F-SiO_2_) nanoparticles on a stainless steel mesh. Utilizing the photocatalytic mesh, the time-dependent evolution of the water-rich permeate flux as a result of photocatalytic degradation of the oil was studied under the visible light illumination. A mathematical model was developed that can relate the photocatalytic degradation of the organic substances deposited on a mesh surface to the evolution of the permeate flux. This model was established by integrating the Langmuir–Hinshelwood kinetics for photocatalysis and the Cassie–Baxter wettability analysis on a chemically heterogeneous mesh surface into a permeate flux relation. Consequently, the time-dependent water-rich permeate flux values are compared with those predicted by using the model. It is found that the model can predict the evolution of the water-rich permeate flux with a goodness of fit of 0.92.

## Introduction

With growing environmental awareness and tighter regulations, there is an increase in investments for developing water remediation technologies^1–8^. Membrane-based technologies are attractive because they are relatively energy-efficient and versatile to different effluents generated in industrial processes^9–12^. One of the primary challenges of membranes is fouling by dissolved or suspended organic substances that can get adsorbed on the membrane surface or pore walls^13,14^. This results in a decrease in the permeability and can eventually shorten the membrane’s life cycle^14–16^. Therefore, membrane-based remediation technologies often involve prefiltration to remove the suspended or dissolved substances^17^. Also, membranes are periodically subjected to cleaning processes such as backwashing, forward flushing, and chemical treatment to remove the surface-deposited contaminants^18,19^. While these methodologies are effective and widely employed in real applications, they can irreversibly degrade membrane’s performance over time^20,21^.

Manipulating the membrane’s wettability has been reported as an alternative to enhance its fouling resistance^22–25^. For example, membranes with hydrophilic (i.e., water contact angle < 90°) or superhydrophilic (i.e., water contact angle $\approx$ 0°) wettability can prevent adsorption of the organic substances (e.g., oil) to the surface by allowing water to form a thin film^26–28^. Also, these membranes can exhibit selective permeation for water while repelling oils, which enables separation of oil–water mixtures with a high separation efficiency^29^. In comparison, membranes possessing lower solid surface energy (*γ*_sv_) can repel the organic substances without needing for a water film^30–32^. We^6^ and others^26–28^ have reported fouling-resistant membranes that can separate oil–water mixtures with an insignificant decline in the permeate flux by combining hydrophilic (or superhydrophilic) wettability along with lower solid surface energy.

Membranes with selective wettability have been incorporated with photocatalytic materials (e.g., TiO_2_^6,33^, N-TiO_2_^34^, α-Fe_2_O_3_^16,35^, Fe_3_O_4_^36^, WO_3_^37^, ZnO^38^, BiVO_4_^7^, α-FeOOH^39^, MoO_3_^40^, Co_3_O_4_^41^, Gd_2_ZnMnO_6_/ZnO^42^) that can degrade the organic substances deposited on the surface upon light illumination. These membranes have demonstrated that they can oxidize (or reduce) the organic substances either dissolved in a liquid (e.g., water) or adsorbed on the membrane surface when irradiated by light with an energy higher than their bandgap energy^43,44^. This can clean the membrane’s surface and purify the permeate. Moreover, these photocatalytic membranes can recover the water-rich permeate flux upon light illumiation after being fouled by organic substances. For example., Zhang et al.^45^ demonstrated in situ recovery of the water-rich permeate flux utilizing a nitrogen doped TiO_2_ coated membrane under visible light illumination. Guo et al.^46^ fabricated a photocatalytic membrane by utilizing BiOBr/Ag nanoparticles that can degrade organic dyes (e.g., methylene Blue, crystal Violet, acid Red 18, and acid Yellow 36) upon UV light illumination and recover the water-rich permeate flux. Liu et al.^47^ fabricated a PVDF-Ni-ZnO composite membrane and demonstrated in situ photocatalysis-driven recovery of the water-rich permeate flux during the filtration of an aqueous solution dissolved with organic substances (e.g., humic acid, sodium alginate, bovine serum albumin). Recently, we^3,6^ developed photocatalytic membranes by coating a commercial filter with a mixture of visible light-active iron-doped TiO_2_ or nitrogen-doped TiO_2_ and perfluorosilane-grafted SiO_2_. These membranes have demonstrated in situ recovery of the water-rich permeate flux upon visible light illumination during oil–water separation.

An increase in the permeate flux upon light illumination has been attributed to the photocatalytic degradation of the organic substances deposited on the membrane surface^27,48,49^. Also, such photocatalytic membranes have demonstrated that they exhibit a time-dependent evolution of the surface chemistry heterogeneity (e.g., clean and contaminated regions) upon light illumination which can be quantitatively described by the contact angle measurements^6,22,50–52^. To our knowledge, quantitative relationships of the evolution of surface chemistry heterogeneity on a membrane surface and that of permeate flux upon visible light illumination are lacking. Establishing such a relation is critical to understand both membrane fouling and photocatalytic cleaning mechanisms, which enables one to design a separation membrane with tailored performance.

Based on these findings, herein, we conducted experimental analysis on the effect of wettability and photocatalyis on the permeate flux through a photocatalytic material-coated stainless steel mesh and developed a mathematical relation between them under visible light illumination. For this, we fabricated a photocatalytic mesh utilizing a stainless steel mesh coated with nitrogen-doped TiO_2_ (N-TiO_2_) and perfluorosilane-grafted SiO_2_ (F-SiO_2_) nanoparticles mixture. A mathematical model was derived by integrating the Langmuir–Hinshelwood kinetic model of photocatalysis^22,30^ and the Cassie–Baxter analysis of the contact angles for water on a photocatalytic surface^53^ into a permeate flux relation^54^. The mathematical model was then utilized to predict the water-rich permeate flux through the photocatalytic mesh during visible light illumination. The accuracy of the predicted flux values was then validated by comparing with the experimentally acquired results.

## Results and discussion

### Photocatalytic mesh fabrication and under-oil water wettability

A mixture of nitrogen-doped TiO_2_ (N-TiO_2_) and perfluorosilane-grafted SiO_2_ (F-SiO_2_) nanoparticles was utilized to fabricate a visible light-active photocatalytic mesh (see “Methods”). Please note that the synthesis of a mixture of N-TiO_2_ and F-SiO_2_ nanoparticles (i.e., N-TiO_2_/F-SiO_2_) was reported in previous work^6^ which demonstrated selective wettability for water over oil (i.e., hydrophilic and oleophobic wettability). Briefly, a dispersion of N-TiO_2_/F-SiO_2_ in deionized (DI) water (solute concentration = 10 wt%) was sprayed onto a stainless steel (SS) 316 Twill Dutch weave mesh (SS mesh) for one minute. Note that the SS mesh was pre-treated with an ultraviolet (UV)-curable adhesive. Here, we utilized dispersions with varied N-TiO_2_ concentrations in the N-TiO_2_/F-SiO_2_ mixture (i.e., 0, 25 wt%, 50 wt%, 75 wt%, and 100 wt%). Subsequently, the SS mesh was illuminated by a long-wavelength UV light (100 W, λ = 365 nm) for 5 min to completely cure the adhesive. Finally, the resulting mesh was thoroughly rinsed with ethanol and DI water.

**Figure 1:**
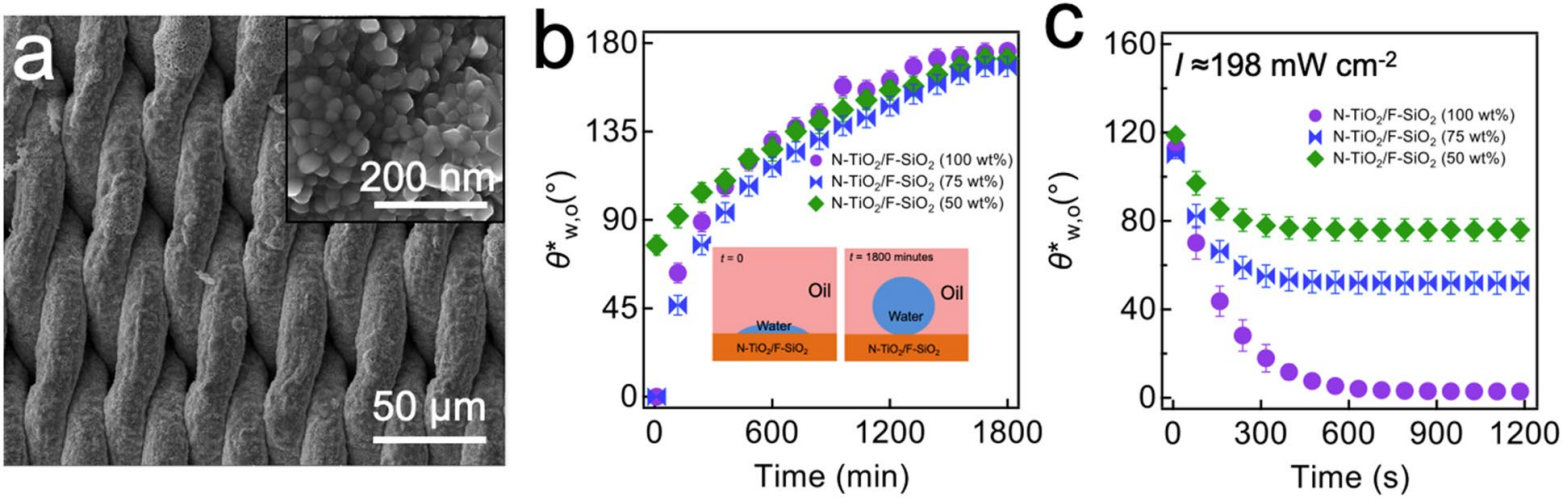
N-TiO_2_/F-SiO_2_ coated mesh surface morphology and evolution of water wettability under oil. **(a)** Scanning electron microscopy (SEM) image of stainless steel (SS) 316 Twill Dutch weave mesh coated with N-TiO_2_/F-SiO_2_ (50 wt%). The inset shows a higher magnification SEM image of the mesh surface. **(b)** The measured apparent contact angles for water on the mesh surfaces coated with N-TiO_2_/F-SiO_2_ mixture with various N-TiO_2_ concentrations (50 wt%, 75 wt%, and 100 wt%) that are submerged in an oil (n-hexadecane) bath as a function of submerging time. The inset images illustrate schematics of the time-dependent evolution of the water contact angles on a mesh surface submerged in oil. **(c)** The measured apparent contact angles for water on the mesh surfaces coated with N-TiO_2_/F-SiO_2_ mixture with various N-TiO_2_ concentrations (50 wt%, 75 wt%, and 100 wt%) while being illuminated by visible light (Intensity (*I*) = 198 mW cm^−2^). Note that all meshes were precontaminated by oil for 600 min.

Figure 1a demonstrates a scanning electron microscopy (SEM) image of a SS mesh coated with N-TiO_2_/F-SiO_2_ mixture that includes 50 wt% N-TiO_2_ (i.e., N-TiO_2_/F-SiO_2_ (50 wt%)). The mesh surface was evenly coated with N-TiO_2_/F-SiO_2_ nanoparticles showing a hierarchical structure (i.e., surface texture with two or more length scales^55^) with a root mean square (RMS) surface roughness^3^ of 0.75 $\pm$ 0.03 $\upmu$m (see “Methods”). Further, the nominal pore size of the mesh was measured as 0.40 $\pm$ 0.03 $\upmu$m after coating with N-TiO_2_/F-SiO_2_ mixture (see “Methods”). Please note that the mesh exhibited mechanical robustness against external stress because the cured adhesive can securely hold nanoparticles on the mesh surface (Supporting Information (SI) Sect. 1).

We^6^ have demonstrated that a commercial filter surface coated with N-TiO_2_/F-SiO_2_ mixture can exhibit varying wettability for water and oil depending on the composition of N-TiO_2_ and F-SiO_2_ (SI Sect. 2). Here, we choose SS meshes exhibiting selective wettability for water over oil in air (i.e., hydrophilic and oleophobic wettability); those coated with 50 wt% or 75 wt% N-TiO_2_ (i.e., N-TiO_2_/F-SiO_2_ (50 wt%) and N-TiO_2_/F-SiO_2_ (75 wt%), respectively). Note that SS meshes coated with N-TiO_2_/F-SiO_2_ (25 wt%) or N-TiO_2_/F-SiO_2_ (0) are excluded in this study because they exhibit in air omniphobic wettability (i.e., hydrophobic and oleophobic wettability) making them less suitable for oil–water separation.

### Time-dependent change of mesh surface wettability submerged in oil

When a hydrophilic (or superhydrophilic) surface is fouled by oil, it often exhibits an increase in the water contact angles^22^. To study the fouling behavior, we submerged our coated mesh in an oil (n-hexadecane) bath and measured the underoil apparent contact angles for water (θ^*^_w,o_) as a function of submerging time. The results show that a mesh coated with a higher concentration of N-TiO_2_ shows a steeper increase in the values of θ^*^_w,o_ (Fig. 1b). For example, a mesh coated with N-TiO_2_/F-SiO_2_ (100 wt%) shows θ^*^_w,o_ = 97° ± 3° while those coated with N-TiO_2_/F-SiO_2_ (75 wt%) and N-TiO_2_/F-SiO_2_ (50 wt%) exhibits 88° ± 3° and 107° ± 3°, respectively, at *t* = 300 min. Please note that the values of θ^*^_w,o_ on as-prepared meshes coated with N-TiO_2_/F-SiO_2_ (100 wt%) and N-TiO_2_/F-SiO_2_ (75 wt%) were zero while we measured θ^*^_w,o_ = 79° ± 3° on an as-prepared mesh coated with N-TiO_2_/F-SiO_2_ (50 wt%). Such a transition to underoil hydrophobicity (i.e., θ^*^_w,o_ > 90°) can be attributed to an increase in the area fraction of the oil adsorbed region on the coated mesh surface which lowers the solid surface energy^22,29^. Note that the θ^*^_w,o_ values became constant at 176° ± 2°, 171° ± 4°, and 178° ± 2° on a mesh coated with N-TiO_2_/F-SiO_2_ (50 wt%), N-TiO_2_/F-SiO_2_ (75 wt%), and N-TiO_2_/F-SiO_2_ (100 wt%), respectively, at *t* = 1800 min.

When an oil-contaminated photocatalytic mesh surface is illuminated by light, it can exhibit a conversion to underoil hydrophilic (or superhydrophilic) wettability due to photocatalytic degradation of the surface adsorbed oil molecules which can lead to an increase in the area fraction of clean (i.e., high solid surface energy) regions^22,29^. We conducted in situ measurements for the θ^*^_w,o_ values on our mesh under visible light illumination (*I* ≈ 198 mW cm^-2^). All meshes were precontaminated with oil for 600 min. Upon the onset of visible light illumination, the θ^*^_w,o_ values started to rapidly decrease and reached constant values after *t* ≈ 900 s (i.e., ≈15 min, see Fig. 1c). Note that a mesh coated with a N-TiO_2_/F-SiO_2_ mixture with a higher concentration of N-TiO_2_ exhibits a rapider decrease in the θ^*^_w,o_ values. For example, a mesh coated with N-TiO_2_/F-SiO_2_ (75%) showed θ^*^_w,o_ = 51° ± 3° whereas that coated with N-TiO_2_/F-SiO_2_ (50%) exhibited θ^*^_w,o_ = 75° ± 3° at *t* ≈ 900 s. Note that a mesh coated with N-TiO_2_/F-SiO_2_ (100%) can completely recover its inherent hydrophilic wettability. We also demonstrated that visible light illumination with a higher intensity can result in a rapider change in the θ^*^_w,o_ values (SI Sect. 3).

### Evolution of the water-rich permeate flux

**Figure 2:**
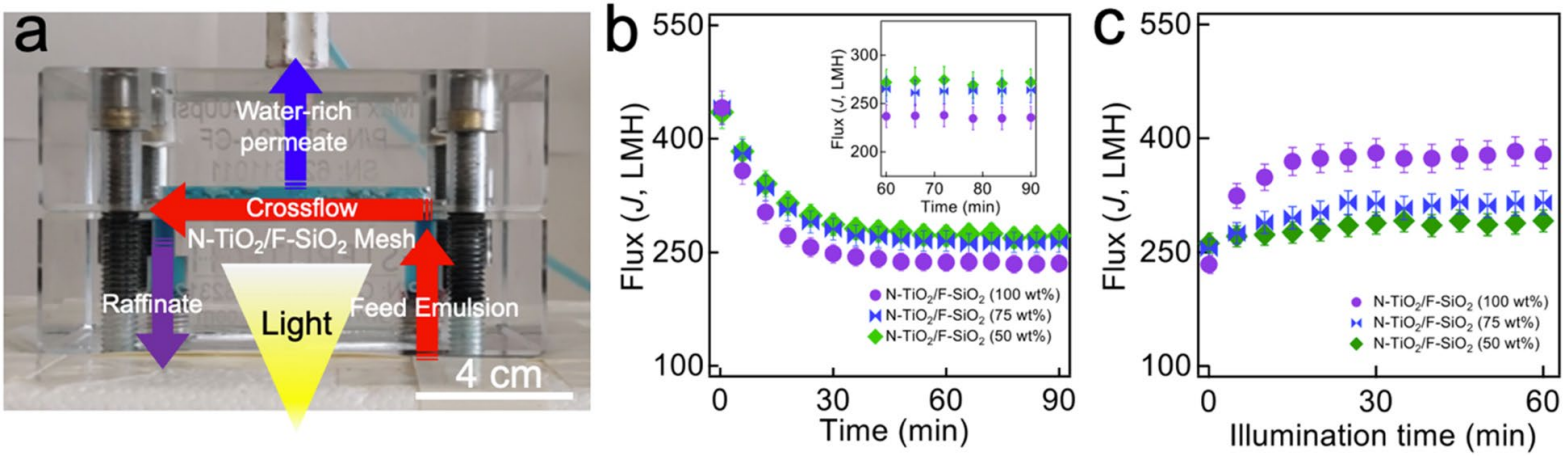
Evolution of water-rich permeate flux during continuous cross-flow separation of oil–water emulsion upon visible light illumination. **(a)** Photograph of the apparatus that enables continuous cross-flow separation of an oil–water mixture and in situ photocatalysis. (water is dyed blue and oil is dyed red). **(b)** The measured flux values (*J*) of the water-rich permeate through the mesh coated with various N-TiO_2_ concentrations (50 wt%, 75 wt%, and 100 wt%) of N-TiO_2_/F-SiO_2_ mixture during separation of SDS-stabilized n-hexadecane-in-water emulsion (1:9 n-hexadecane:water volumetric ratio). The inset shows zoomed-in flux values in the time interval of 60–90 min. **(c)** The measured water-rich permeate flux values (*J*) while being illuminated by visible light (*I* ≈ 198 mW cm^−2^).

The selective wettability for water over oil, along with its photocatalytic degradation capability enable our mesh to exhibit enhanced resistance to oil fouling and photocatalytic cleaning of the surface under light illumination when subjected to an oil–water mixture^22,29^. A continuous cross-flow separation apparatus^6,56^ was utilized to conduct oil–water separation and in situ photocatalysis (Fig. 2a). Here, a feed oil–water mixture is continuously fed by a plunger pump and the water-rich permeate passes through the mesh and collected in a container. An n-hexadecane-in-water emulsion (1:9 volumetric ratio, n-hexadecane:water) stabilized by a surfactant (sodium dodecyl sulfate, SDS) was utilized (see “Methods”). Note that a mesh was prewetted by SDS-dissolved water (SDS concentration = 0.015 wt% with respect to water weight) for 30 min (flow rate = 2.0 L s^−1^ ± 0.2 L s^−1^) to obtain a constant flux (*J*_o_) for the water-rich permeate before introducing a feed emulsion. The transmembrane pressure (∆*p*, i.e., the difference in pressure at two opposite sides of the mesh) was maintained at ∆*p* = 13.0  ± 0.5 kPa for prewetting process.

When a feed oil-in-water emulsion was introduced (∆*p* = 13.0  ± 0.7 kPa and flow rate = 2.0 L s^−1^ ± 0.1 L s^−1^), the flux values (*J*) for the water-rich permeate rapidly decreased, which can be primarily attributed to fouling of the mesh surface by oil (Fig. 2b)^3,6,15,39,57^. The results show that a mesh coated with N-TiO_2_/F-SiO_2_ with a higher concentration of N-TiO_2_ exhibits a steeper decrease in the *J* values that eventually reaches lower values at *t* ≈ 90 min. For example, a mesh coated with N-TiO_2_/F-SiO_2_ (50 wt%) exhibits *J* ≈ 261 L m^−2^ h^−1^ (LMH) while that coated with N-TiO_2_/F-SiO_2_ (75 wt%) shows *J* ≈ 253 LMH at *t* ≈ 90 min. Given that the *J*_o_ values were ≈435 LMH and ≈441 LMH for a mesh coated with 50 wt% N-TiO_2_ and 75 wt% N-TiO_2,_ respectively, they correspond to ≈60% and ≈57% of the respective *J*_o_ values. Please note that the *J* values were measured by a relation^58^, *J* = ∆*m*(A*ρ*∆*t*)^−1^. Here, ∆*m* represents the change in the water-rich permeate mass for a given time interval (∆*t* = 5 min), *A* is the projected area of the mesh (*A* = 42 cm^2^), and *ρ* is the density of the permeate (*ρ* ≈ 0.998 g cm^−3^). Also note that the oil concentration in the water-rich permeate remains very low (i.e., < 0.2 wt%) despite a decrease in water-rich permeate flux (SI Sect. 4).

When the water-rich permeate flux exhibited a constant value at *t* ≈ 90 min (i.e., illumination time, *t*_i_ = 0), we started illuminating the mesh surface with visible light (*I* ≈ 198 mW cm^−2^) while the mesh was continuously subjected to a fresh feed emulsion. Figure 2c shows that the *J* values start to increase upon visible light illumination. This indicates cleaning of the oil-contaminated mesh surface which consequently results in a lower breakthrough pressure (i.e., a minimum applied pressure at which the water permeates through the mesh) for the water-rich permeate^3,6^. Also, a mesh coated with higher concentration of N-TiO_2_ exhibted a higher recovery rate of the permeate flux values. For example, a mesh coated with N-TiO_2_/F-SiO_2_ (50 wt%) showed *J* ≈ 291 LMH after 60 min of visible light illumination (i.e., *t*_i_ = 60 min), whereas that coated with N-TiO_2_/F-SiO_2_ (75 wt%) showed *J* ≈ 315 LMH. This corresponds to 17% and 30% recovery.

### Mathematical representation of the permeate flux kinetics

It is postulated that the extent of permeate flux recovery upon light illumination depends on various experimental parameters that include the incident light intensity (*I*), the active surface area (*A*) of the N-TiO_2_/F-SiO_2_ coating, and the photocatalytic degradation rate (*k*_p_)^59^. Here, we develop a mathematical model that can describe the time-dependent evolution of the water-rich permeate flux through a mesh coated with N-TiO_2_/F-SiO_2_ upon visible light illumination.

When a photocatalytic mesh surface is subjected to oil submerged in water while being illuminated by visible light, three chemical reactions can take place: adsorption, desorption, and photocatalytic degradation of oil molecules^30,60–62^. We^22^ recently showed that these reactions obey the first-order kinetics. Assuming that N-TiO_2_ is photocatalytic^63–65^ while F-SiO_2_ is not^6^, the following differential equation can be obtained which describes a time-dependent photocatalysis-driven evolution of the area fraction of the mesh surface contaminated with oil (*f*_c_(*t*_i_):

1 $$\frac{d}{dt}{f}_{\rm{c}}\left({t}_{i}\right)={f}_{\rm{T}}\times \frac{d}{dt}{f}_{\rm{c}(\rm{T})}\left({t}_{i}\right)+{f}_{\rm{F}}\times \frac{d}{dt}{f}_{\rm{c}(\rm{F})}\left({t}_{i}\right)$$

where *f*_(T)_ and *f*_(F)_ are the area fraction of N-TiO_2_ and F-SiO_2_, respectively. The subscripts T and F symbolize N-TiO_2_ and F-SiO_2_, respectively. Solving Eq. (1) by substituting $\frac{d}{dt}{f}_{\rm{c}(\rm{T})}\left({t}_{i}\right)= {k}_{\rm{a}(\rm{T})}{f}_{\rm{nc}(\rm{T})}-{k}_{\rm{d}\left(\rm{T}\right)}{f}_{\rm{c}\left(\rm{T}\right)}-{k}_{\rm{p}(\rm{T})}{f}_{\rm{c}(\rm{T})}$ and $\frac{d}{dt}{f}_{\rm{c}(\rm{F})}\left({t}_{i}\right)= {k}_{\rm{a}(\rm{F})}{f}_{\rm{nc}(\rm{F})}-{k}_{\rm{d}\left(\rm{F}\right)}{f}_{\rm{c}\left(\rm{F}\right)}$, where *k*_a_*, k*_d_, and *k*_p_ are the rate constant values for adsorption, desorption, and photocatalytic degradation of oil, respectively, on a particular phase (e.g., N-TiO_2_ or F-SiO_2_), and ${f}_{\rm{nc}(\rm{T})}$ = $1-{f}_{\rm{c}(\rm{T})}$ and ${f}_{\rm{nc}(\rm{F})}$ = $1-{f}_{\rm{c}(\rm{F})}$ (i.e., non-contaminated area fraction of each phase, *f*_nc(T)_ or *f*_nc(F)_) results in:

2 $${f}_{\rm{c}}({t}_{i})=\left[ \frac{{k}_{\rm{a}(\rm{T})}}{{K}_{(\rm{T})}}-\left(\frac{{k}_{\rm{a}(\rm{T})}}{{K}_{(\rm{T})}}-{f}_{\rm{c}(\rm{T})}\left({t}_{i}=0\right)\right){\rm{e}}^{-\left({K}_{(\rm{T})}\right){t}_{i}}\right]\times {f}_{(\rm{T})}+\left[\frac{{k}_{\rm{a}(\rm{F}) }}{{K}_{(\rm{F})}}-\left(\frac{{k}_{\rm{a}(\rm{F}) }}{{K}_{(\rm{F})}}-{f}_{c(\rm{F})}\left({t}_{i}=0\right)\right){\rm{e}}^{-\left({K}_{(\rm{F})}\right){t}_{i}}\right]\times {f}_{(\rm{F})}$$

where *f*_c(T)_(*t*_i_ = 0) and *f*_c(F)_(*t*_i_ = 0) are the initial area fraction of the contaminated regions for N-TiO_2_ and F-SiO_2_ at the start of visible light illumination, respectively, which are assumed to be zero. Here *K*_(T)_ and *K*_(F)_ are defined as ${{K}_{(\rm{T})}= k}_{\rm{a}(\rm{T})}+{k}_{\rm{d}(\rm{T})}+{k}_{\rm{p}(\rm{T})}$ and ${{K}_{(\rm{F})}= k}_{\rm{a}(\rm{F})}+{k}_{\rm{d}(\rm{F})}$, respectively.

The time-dependent flux of the water-rich permeate under visible light illumination (*J*(*t*_i_)) can be written as^54^:

3 $$J\left({t}_{i}\right)=\Delta P/ \left[\left({r}_{m}+{R}_{c}/A(1-{f}_{c}({t}_{i})\right)\mu \right]$$

where *∆P* and *A* are the transmembrane pressure and the total surface area of the mesh, respectively. *r*_m_ and *R*_c_ are the resistance per unit area of the mesh to the permeation of the water-rich permeate originated from the mesh itself and oil contamination, respectively. *µ* is the dynamic viscosity of the water-rich permeate^66^ ($\approx$ 0.953 mPa-s). By substituting ${f}_{\rm{c}}\left({t}_{i}\right)$ in Eq. (3), we obtain the following equation:

4 $$J\left({t}_{i}\right)=\Delta P/\left[\left\{{r}_{m}+\frac{{R}_{c}}{A}\times \frac{1}{\left(1-\left(\begin{array}{c} \\ {f}_{(\rm{F})}\left[ \frac{{k}_{\rm{a}\left(\rm{T}\right)}}{{K}_{(\rm{T})}}-\left(\frac{{k}_{\rm{a}\left(\rm{T}\right)}}{{K}_{(\rm{T})}}-{f}_{\rm{c}\left(\rm{T}\right)}\left({t}_{i}=0\right)\right){\rm{e}}^{-\left({K}_{(\rm{T})}\right){t}_{i}}\right]\times {f}_{(\rm{T})}+\left[ \frac{{k}_{\rm{a}(\rm{F})}}{{K}_{(\rm{F})}}-\left(\frac{{k}_{\rm{a}(\rm{F})}}{{K}_{(\rm{F})}}-{f}_{\rm{c}(\rm{F})}\left({t}_{i}=0\right)\right){\rm{e}}^{-\left({K}_{(\rm{F})}\right){t}_{i}}\right]\times {f}_{(F)}\end{array}\right)\right)}\right\}\mu \right]$$

This equation describes the time-dependent evolution of the water-rich permeate flux through the mesh subjected to oil upon illumination by visible light. Please note that the details of calculations for the variables (e.g., *r*_m_, *R*_c_, *f*_c(T)_, and *f*_c(F)_) in Eq. (4) are included in SI Sect. 5.

### Extraction of rate constants and prediction of the flux

The values of *k*_a_ and *k*_d_ can be determined by analyzing the time-dependent evolution of the θ^*^_w,o_ values in dark, whereas the *k*_p_ values can be determined under visible light illumination. In our recent work^22^, we demonstrated that these rate constants (*k*_a_, *k*_d_, and *k*_p_) can be related to the measured θ^*^_w,o_, values on a photocatalytic surface. Here, we develop a new relation by considering that our mesh surface is heterogenous consisting of photocatalytic N-TiO_2_ and inert (i.e., non-catalytic) F-SiO_2_. By integrating the Langmuir–Hinshelwood kinetics for photocatalysis^22,30^ and the Cassie–Baxter wettability analysis^53^ on a chemically heterogeneous mesh surface, we can obtain a relation given as:

5 $$\rm{cos}{\theta }_{\rm{w},\rm{o}}^{*}=1-2\left(\left[ \frac{{k}_{\rm{a}\left(\rm{T}\right)}}{{K}_{\left(\rm{T}\right)}}-\left(\frac{{k}_{\rm{a}\left(\rm{T}\right)}}{{K}_{\left(\rm{T}\right)}}-{f}_{\rm{c}\left(\rm{T}\right)}\left({t}_{i}=0\right)\right){\rm{e}}^{-\left({K}_{\left(\rm{T}\right)}\right){t}_{i}}\right]\times {f}_{\left(\rm{T}\right)}+\left[\frac{{k}_{\rm{a}\left(\rm{F}\right)}}{{K}_{\left(\rm{F}\right)}}-\left(\frac{{k}_{\rm{a}\left(\rm{F}\right)}}{{K}_{\left(\rm{F}\right)}}-{f}_{c\left(\rm{F}\right)}\left({t}_{i}=0\right)\right){\rm{e}}^{-\left({K}_{\left(\rm{F}\right)}\right){t}_{i}}\right]\times {f}_{\left(\rm{F}\right)}\right)$$

**Figure 3:**
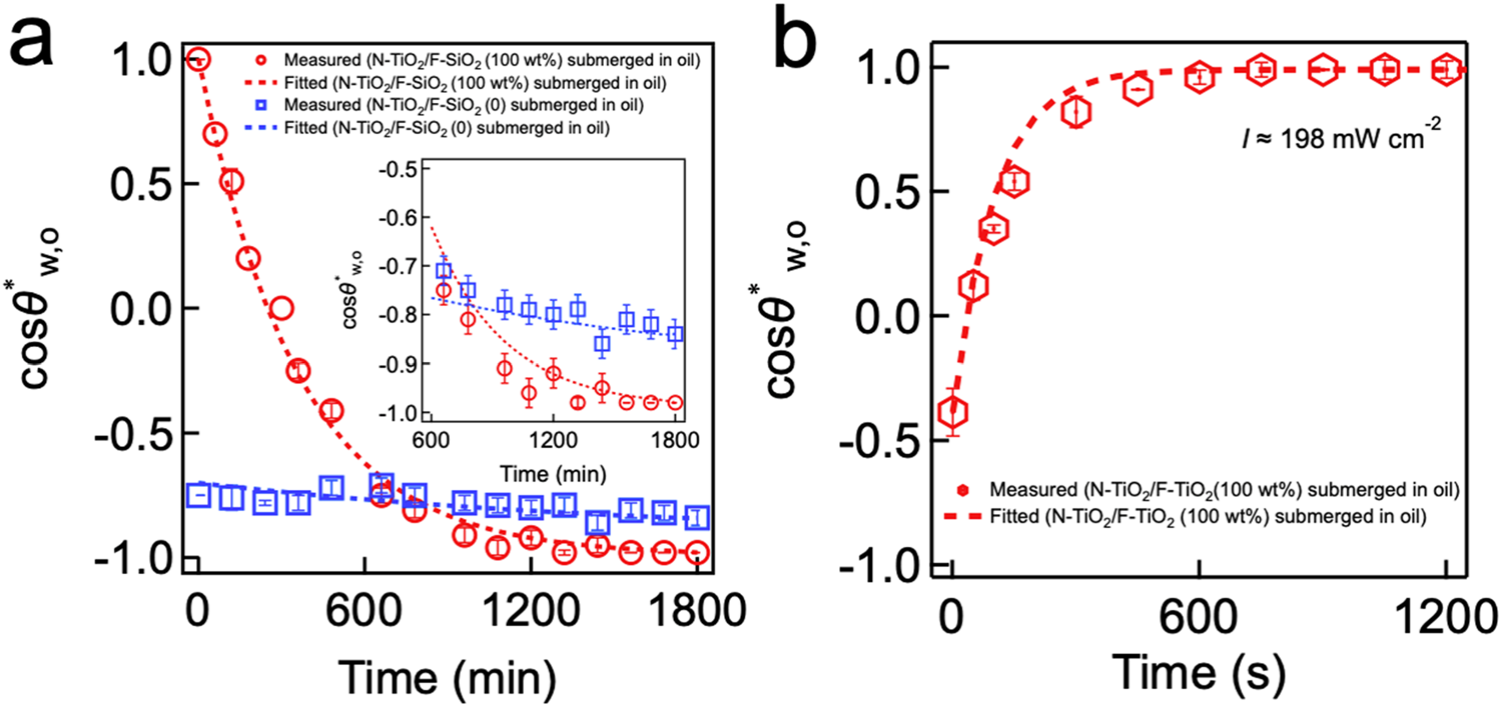
Extraction of adsorption, desorption, and photocatalytic rate constants. **(a)** Plots of the cosine values of the measured apparent water contact angle (θ^*^_w,o_) on an N-TiO_2_ surface and an F-SiO_2_ surface submerged in oil (n-hexadecane) as a function of submerging time. The values of *k*_a_ and *k*_d_ for oil on N-TiO_2_ and F-SiO_2_ surfaces were determined by fitting Eq. (5). Inset: Zoomed-in plot of the cosθ^*^_w,o_ data. **(b)** A plot of the cosine values of the measured apparent water contact angle (θ^*^_w,o_) on an N-TiO_2_ surface submerged in oil as a function of visible light illumination (*I* ≈ 198 mW cm^-^^2^) time. The *k*_p_ value for N-TiO_2_ was determined by fitting Eq. (5).

The values of *k*_a*,*_* k*_d_, and *k*_p_ for a given phase (e.g., N-TiO_2_ or F-SiO_2_) can be obtained by fitting Eq. (5) to the cosine values of the experimentally measured θ^*^_w,o_ values. Figure 3a shows a plot of the cosine values of the experimentally measured θ^*^_w,o_ on N-TiO_2_ and F-SiO_2_ surfaces submerged in oil as a function of submerging time. Note that we utilized the θ^*^_w,o_ values of N-TiO_2_/F-SiO_2_ (100%) shown in Fig. 1b. We obtained the values of *k*_a_ and *k*_d_ for oil on an N-TiO_2_ surface as *k*_a(T)_ = 4.65 × 10^−5^ s^−1^ and *k*_d(T)_ = 2.3 × 10^−7^ s^−1^, respectively, while those on an F-SiO_2_ surface were *k*_a(F)_ = 9.54 × 10^−6^ s^−1^ and *k*_d(F)_ = 4.06 × 10^−7^ s^−1^, respectively. Please note that the *k*_a_ value for oil on an F-SiO_2_ surface is an order of magnitude lower than that on an N-TiO_2_ surface which clearly indicates that F-SiO_2_ is more resistant to oil adsorption.

Similarly, the *k*_p_ value can be obtained by fitting Eq. (5) to the cosine values of the experimentally measured time-dependent θ^*^_w,o_ on a surface that was submerged in oil and placed under visible light illumination (Fig. 3b). Note that the θ^*^_w,o_ values of N-TiO_2_/F-SiO_2_ (100%) shown in Fig. 1c were utilized. The *k*_p(T)_ value for N-TiO_2_ is 9.8 × 10^−3^ s^−1^ which is two orders of magnitude higher than the *k*_a(T)_ value (*k*_a(T)_ = 4.65 × 10^−5^ s^-1^). Thus, it can be inferred that N-TiO_2_ can rapidly clean itself upon visible light illumination despite being submerged in oil. Note that the *k*_p_ value for F-SiO_2_ surface (i.e., *k*_p(F)_) is zero. The *k*_*p*_ values for N-TiO_2_ obtained by using different visible light intensities are also included in SI Sect. 6.

**Figure 4:**
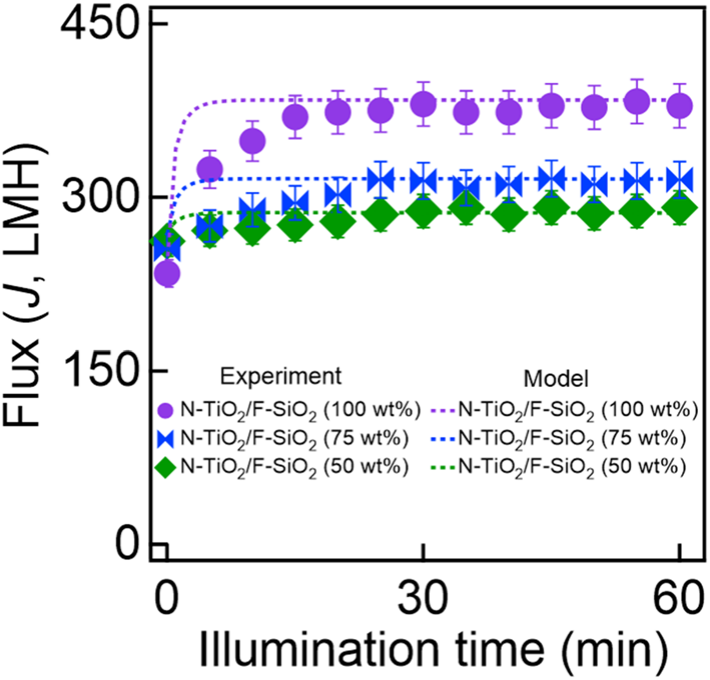
The measured and the predicted values of water-rich permeate flux (*J*) by using Eq. (4) through the meshes coated with varied compositions of N-TiO_2_/F-SiO_2_ under visible light illumination (*I* = 198 mW cm^−2^).

Finally, we calculated the *J*(*t*_i_) values for the mesh coated with varied compositions of N-TiO_2_/F-SiO_2_ by using the values of *k*_a*,*_* k*_d,_ and *k*_p_ in Eq. (4) and compared them with the experimentally measured values. Figure 4 shows that they match reasonably well with a goodness of fit equal to 0.92.

## Conclusions

In summary, a photocatalytic mesh with selective wettability for water over oil was developed by coating a mixture of N-TiO_2_/F-SiO_2_ onto a surface of a stainless steel mesh. The mesh was utilized to study the kinetics of the water-rich permeate flux as a result of the photocatalytic degradation of the surface-adsorbed oil under visible light illumination. A mathematical model was derived by integrating the Langmuir–Hinshelwood kinetics of photocatalysis and the Cassie–Baxter wettability analysis on a chemically heterogeneous surface into a permeate flux relation. Finally, this model demonstrated that it can predict the evolution of the water-rich permeate flux through the photocatalytic mesh with a goodness of fit of 0.92. We envision that the outcomes of this study can find applicability in designing and optimizing photocatalytic membranes for multiphase interfacial engineering applications such as oil–water separation.

## Methods

### Synthesis of N-TiO_2_ and F-SiO_2_ nanoparticles

N-TiO_2_ and F-SiO_2_ nanoparticles were synthesized by employing a modified sol–gel method according to the procedures in our previous work^6^. For N-TiO_2_, titanium butoxide (TBOT, 5.0 g) was added dropwise to a mixture of isopropyl alcohol (IPA) and DI water (1:9 volumetric ratio, IPA:DI water). The pH of the solution was adjusted to 2.0 ± 0.1 by adding nitric acid (0.01 M). Subsequently, triethylamine with a molar ratio of 2.0 with respect to TBOT was added dropwise to the solution. After stirring the solution for 12 h at 30 °C, the precipitates were collected by centrifugation and thoroughly rinsed with ethanol and DI water. The product was vacuum dried to obtain N-TiO_2_. For synthesizing F-SiO_2_, tetraethyl orthosilicate (TEOS, 1.0 g) was mixed with a 0.01 M hydrochloric acid in DI water (100 g). 1H,1H,2H,2H-perfluorodecyl trichlorosilane (1.0 g) was then added to the mixture dropwise. The solution was magnetically stirred for 60 min at 60 °C, and the centrifugation was utilized to collect the resulting precipitates. The precipitates were then thoroughly rinsed with ethanol and DI water followed by vacuum drying to obtain F-SiO_2_ nanoparticles.

### Fabrication of photocatalytic mesh with selective wettability for water over oil

Stainless steel (SS) 316 Twill Dutch weave mesh (area = 42 cm^2^) was cleaned with ethanol in an ultrasonic bath for 10 min. The mesh was then dip-coated in a Norland ultraviolet (UV) light-curable optical adhesive (NOA 61) (1.0 wt% in acetone). Subsequently, a dispersion of N-TiO_2_/F-SiO_2_ mixture in DI water (solute concentration = 10 wt%) was sprayed (IWata Eclipse, Anest IWata) onto the adhesive-coated mesh for one minute. The spraying distance and nitrogen gas pressure were maintained at 15 cm and 200 kPa, respectively. The concentrations of N-TiO_2_ nanoparticles in the N-TiO_2_/F-SiO_2_ mixture were 0, 25 wt%, 50 wt%, 75 wt%, and 100 wt%. The mesh was then illuminated by a long-wavelength UV light (100 W, λ = 365 nm, UVA Blak-Ray B100A, Analytikjena) for 5 min to cure the adhesive. Finally, the fabricated photocatalytic mesh was thoroughly rinsed with ethanol and DI water.

### Surfactant-stabilized oil-in-water emulsion

An oil‐in‐water emulsion was prepared by mixing n‐hexadecane and DI water at 1:9 n‐hexadecane:water volumetric ratio. Sodium dodecyl sulfate (SDS) surfactant (0.015 wt% with respect to water) was then added and mixed vigorously to stabilize the emulsion.

### Scanning electron microscopy (SEM)

The surface texture of a mesh coated with N-TiO_2_/F-SiO_2_ nanoparticles was characterized by field-emission scanning electron microscopy (FESEM, FEI Versa 3D). The characterizations were performed at an accelerating voltage of 10 kV.

### Determining the nominal pore size of mesh

Filter retention analysis^3,67^ was utilized to determine the nominal pore size of the mesh. We sequentially fed monodisperse SiO_2_ particles with various diameters to the mesh in the order of the lowest to the highest diameter. We calculated the proportion of the particles retained on the mesh for each diameter according to, %*R* = *M*_R_/*M*_T_, where *M*_R_ and *M*_T_ are the mass of SiO_2_ retained on the mesh and the total mass of that introduced to the mesh, respectively. We assigned the diameter of SiO_2_ as the nominal pore size of the mesh if %*R* exceeds 50% for that particular diameter. Note that we used SiO_2_ particles with diameters of 120, 150, 200, 300, 400, 500, 600, and 750 nm and prepared suspensions in ethanol with a concentration of 50 mg mL^−1^. We measured the %*R* as 66, 69, and 71% with the SiO_2_ possessing a diameter of 400 nm for meshes coated with N-TiO_2_/F-SiO_2_ mixture with 50, 75 and 100 wt% of N-TiO_2_, respectively. Therefore, 400 nm was assigned as the nominal pore size of meshes.

### Contact angle measurement

All contact angle measurements were conducted by utilizing a Rame-´ hart 190-U1 goniometer. About 3 μL of liquids were used during the measurements.

### Visible light intensity measurement

To measure the intensity of the incident visible light on a mesh surface coated with N-TiO_2_/F-SiO_2_, a photometer (Fisherbrand Traceable DualDisplay Lightmeter) was employed. The photometer was placed underneath the top cover of the continuous cross‐flow separation cell and illuminated by the visible light source. Please note that the visible light was illuminated onto the photometer from a distance of ≈5 cm, which is the same as the distance between the light source and the mesh surface during the separation.

### Root mean square (RMS) roughness mesurements

Optical profiler (Veeco Wyko NT 1100) was utilized to measure the root mean square (RMS) surface roughness of coated meshes. The scan rate was set to 50 nm s^−1^. The scanned area was 5 µm × 5 µm.

## Supplementary Information

Supplementary Information.
