# Supplementary material for: Predicting kinetics of water-rich permeate flux through photocatalytic mesh under visible light illumination
Source: Sci Rep. 2021 Oct 26;11:21065. doi: 10.1038/s41598-021-00607-w (PMC8548496; doi:10.1038/s41598-021-00607-w)
Supplement: Supplementary file 1 — Supplementary Information. [file 41598_2021_607_MOESM1_ESM.docx]

Supporting Information

Predicting Kinetics of Water-rich Permeate Flux through Photocatalytic Mesh under Visible Light Illumination

*Bishwash Shrestha*^1‡^*, Mohammadamin Ezazi*^1‡^*, Seyed Vahid Rad*^1^, *and Gibum Kwon*^1^*^,^**

^1^Department of Mechanical Engineering, University of Kansas, Lawrence, Kansas 66045, United States.

*[gbkwon@ku.edu](mailto:gbkwon@ku.edu)

^‡^ Equally contributed

**Section 1. The adhesion strength measurement of N-TiO_2_/F-SiO_2_ nanoparticles to the mesh surface**

Shear stresses exerted by the feed stream on a mesh coated with nanoparticles often causes delamination of them from the surface. We measured the adhesion strength of N-TiO_2_/F-SiO_2_ nanoparticles to the mesh surface by utilizing the standard ASTM D3359 Tape Peel off test.^1^ The mass of the mesh before and after the test was measured by utilizing a high precision scale (Mettler Toledo XS105 DU, precision = 0.01 mg). Note that the mass of the as-purchased mesh was 3,349.5 mg ± 50 mg. The results show that the mass of the mesh coated with N-TiO_2_/F-SiO_2_ nanoparticles utilizing the ultraviolet (UV)-curable adhesive (NOA 61) remains almost unchanged (**Figure**[**S1**](https://onlinelibrary.wiley.com/doi/full/10.1002/gch2.202000009#gch2202000009-fig-0002)). This can be attributed to the cured adhesive which can hold the N-TiO_2_/F-SiO_2_ nanoparticles together and securely bind them to the mesh surface by forming an interlocking structure. In contrast, the mesh coated with N-TiO_2_/F-SiO_2_ nanoparticles without adhesive exhibited $\approx$89±2 % mass loss after the Tape Peel off test.


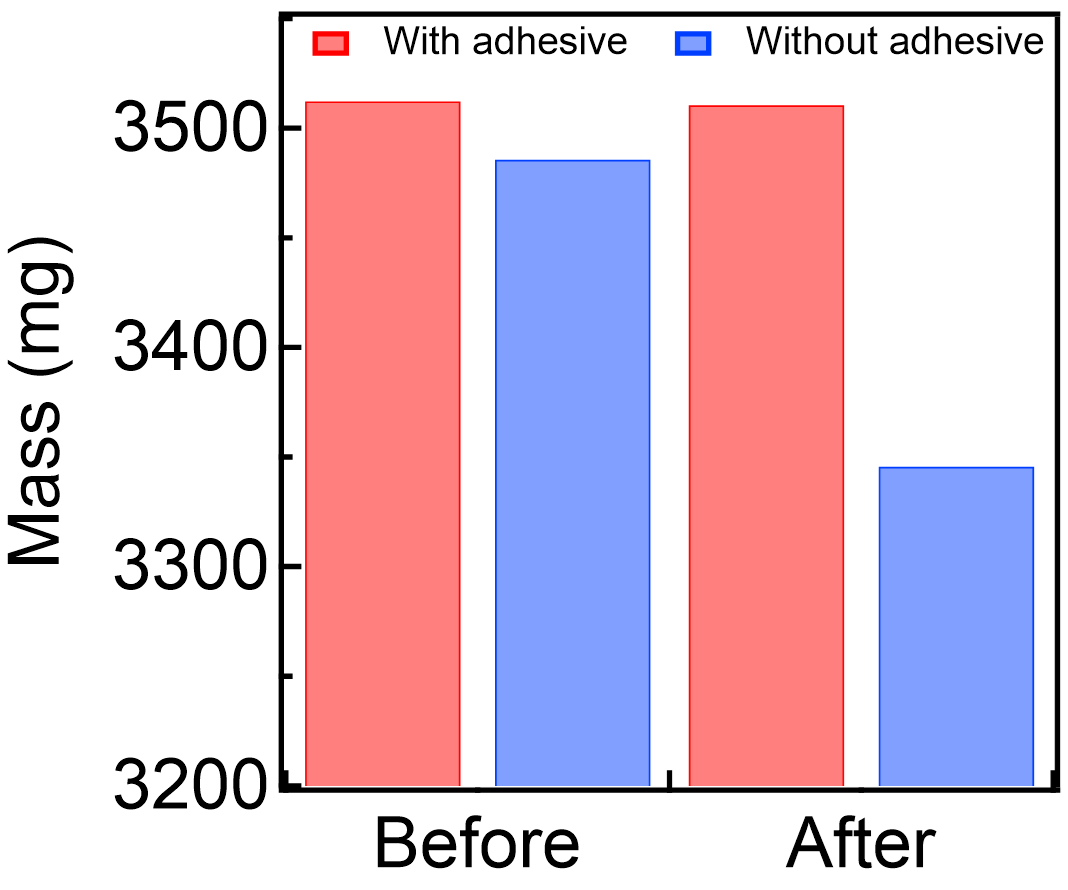


**Figure S1**. The measured mass of the mesh coated with N-TiO_2_/F-SiO_2_ with and without the cured adhesive after the standard Tape Peel off test.

**Section 2. In air and underwater contact angle measurements**

We measured in air apparent advancing (*θ^*^*_adv_) and receding (*θ^*^*_rec_) contact angles of water (*γ*_lv_ = 72.1 mN m^-1^) and oil (n-hexadecane, *γ*_lv_ = 27.5 mN m^-1^) on the mesh surfaces coated with various compositions of N-TiO_2_/F-SiO_2_ (**Figure S2**a). The results indicate that a mesh coated with N-TiO_2_/F-SiO_2_ with a lower N-TiO_2_ concentration exhibits a higher apparent contact angles for both water and oil. When the N-TiO_2_ concentration reached 50 wt%, the apparent contact angles of water becomes lower than oil contact angles (i.e., *θ^*^* _water,adv (in-air)_ = 55°± 2° and *θ^*^* _water,rec (in-air)_ = 20°± 2° while those for oil measured as *θ^*^*_oil,adv (in-air)_ = 97°± 2°, *θ^*^*_oil,rec (in-air)_ = 65°± 2°). Note that a mesh coated with only N-TiO_2_ (i.e., N-TiO_2_/F-SiO_2_ (100 wt%)) exhibits both water and oil apparent contact angles zero.

We also measured the apparent advancing and receding contact angles of oil (n-hexadecane) on the mesh submerged in water (**Figure S2**b). The results indicate that a mesh exhibiting a lower water apparent contact angle in air shows a higher underwater oil apparent contact angle. For example, a mesh coated with N-TiO_2_/F-SiO_2_ (50 wt%) exhibits *θ^*^*_oil,adv (under water)_ = 171°± 5° and *θ^*^*_oil,rec (under water)_ = 165°± 5° while that coated with N-TiO_2_/F-SiO_2_ (25 wt%) shows *θ^*^*_oil,adv (under water)_ = 168°± 5° and *θ^*^*_oil,rec (under water)_ = 163°± 4°.


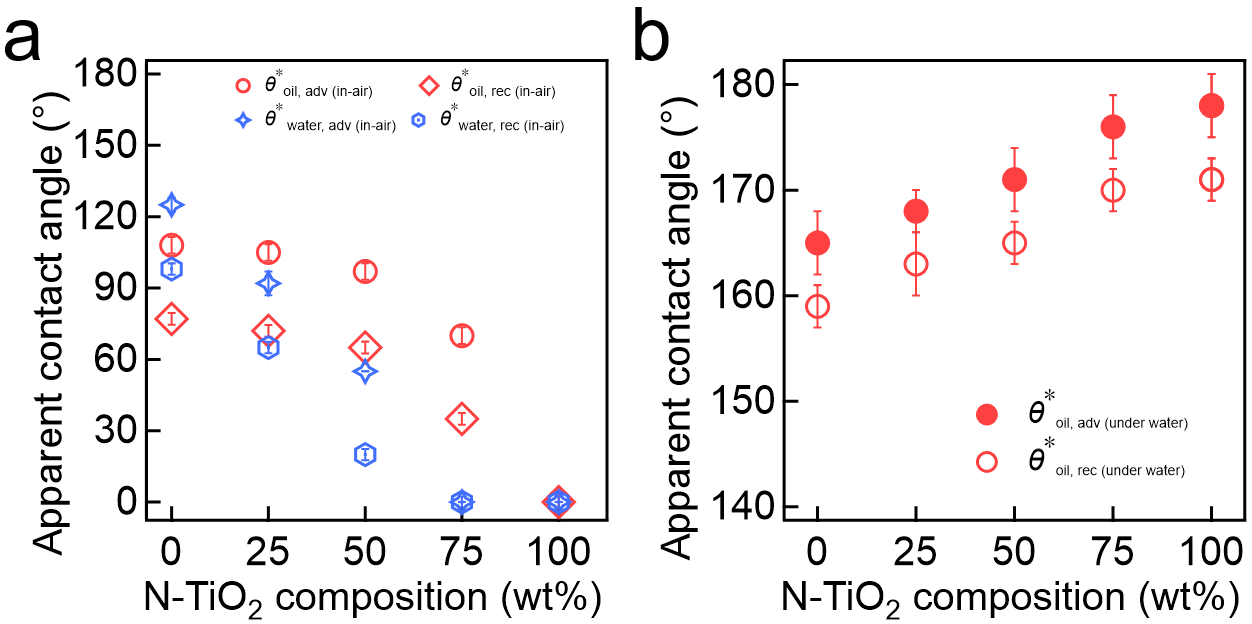


**Figure S2**. (a) The measured in air advancing and receding apparent contact angles for water and oil (n-hexadecane) on the mesh surfaces coated with varied compositions of N-TiO_2_/F-SiO_2_. (b) The measured advancing and receding apparent contact angles of oil (n-hexadecane) on the mesh submerged in water.

**Section 3. Effect of visible light intensity on the apparent water contact angles on a surface submerged in oil**

**Figure S3**a and **S3**b show the time-dependent *θ^*^*_w,o_ values measured on the meshes coated with various compositions of N-TiO_2_/F-SiO_2_ submerged in oil while illuminated by visible light intensity of *I* = 30 mW cm^−2^ and 100 mW cm^−2^, respectively. Please note that we utilized the meshes precontaminated by oil for 600 minutes. The results show that the values of *θ^*^*_w,o_ decrease at a higher rate on a mesh surface illuminated by higher intensity visible light. For example, a mesh coated with N-TiO_2_/F-SiO_2_ (75 wt%) shows a decrease in the value of *θ^*^*_w,o_ to 99°±3° in 600 s when illuminated by light with an intensity of *I* = 30 mW cm^−2^ whereas the *θ^*^*_w,o_ becomes 62°±3° when *I* = 100 mW cm^−2^.


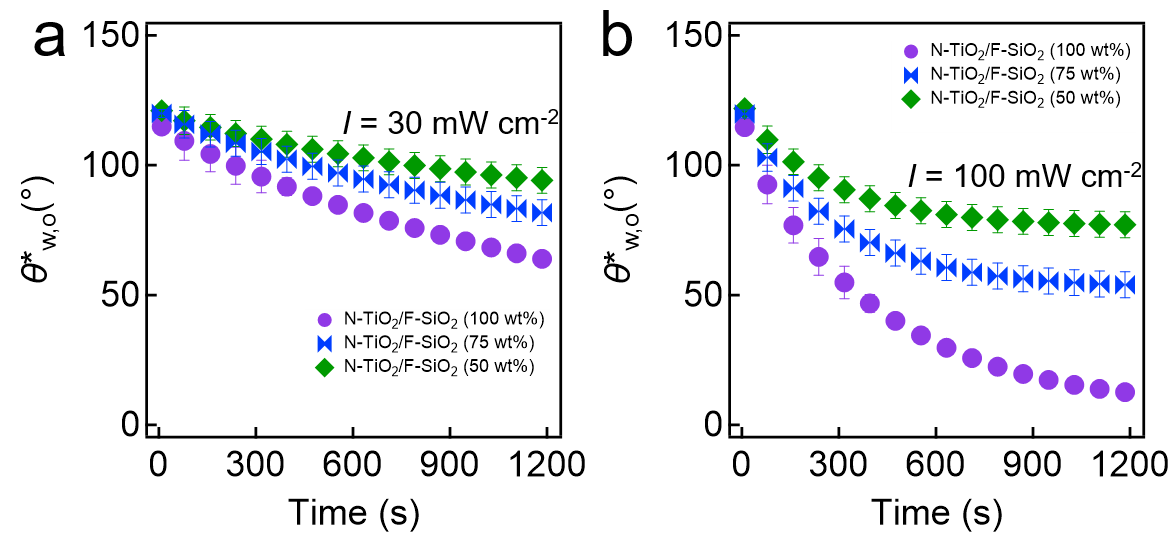


**Figure S3**. (a-b) The measured apparent contact angles of water on mesh surface submerged in oil while being illuminated by visible light with intensity of *I* = 30 mW cm^-2^ (a) and *I* = 100 mW cm^-2^ (b). Note that all meshes were precontaminated by oil for 600 minutes.

**Section 4. Measuring the oil concentration in the water-rich permeate**

Thermogravimetric analyses (TGA, PerkinElmer PYRIS 1) were employed to measure the concentration of oil in the water-rich permeate obtained after the separation of SDS-stabilized n-hexadecane-in-water emulsion. Approximately 10 mg of the water-rich permeate was heated from room temperature ($\approx$22 °C) to 110 °C at a rate of 5 °C min^-1^ followed by maintaining the temperature (110 °C) for 50 minutes. Given that the boiling points of water and oil (n-hexadecane^2^) are 100 °C and $\approx$287 °C, respectively, the remnant can be assumed as pure n-hexadecane. **Figure S4** shows the TGA data of the water-rich permeate through the mesh coated with N-TiO_2_/F-SiO_2_ with varied compositions. The results indicate that the oil concentrations in the water-rich permeate are very low (i.e., < 0.2 wt%).


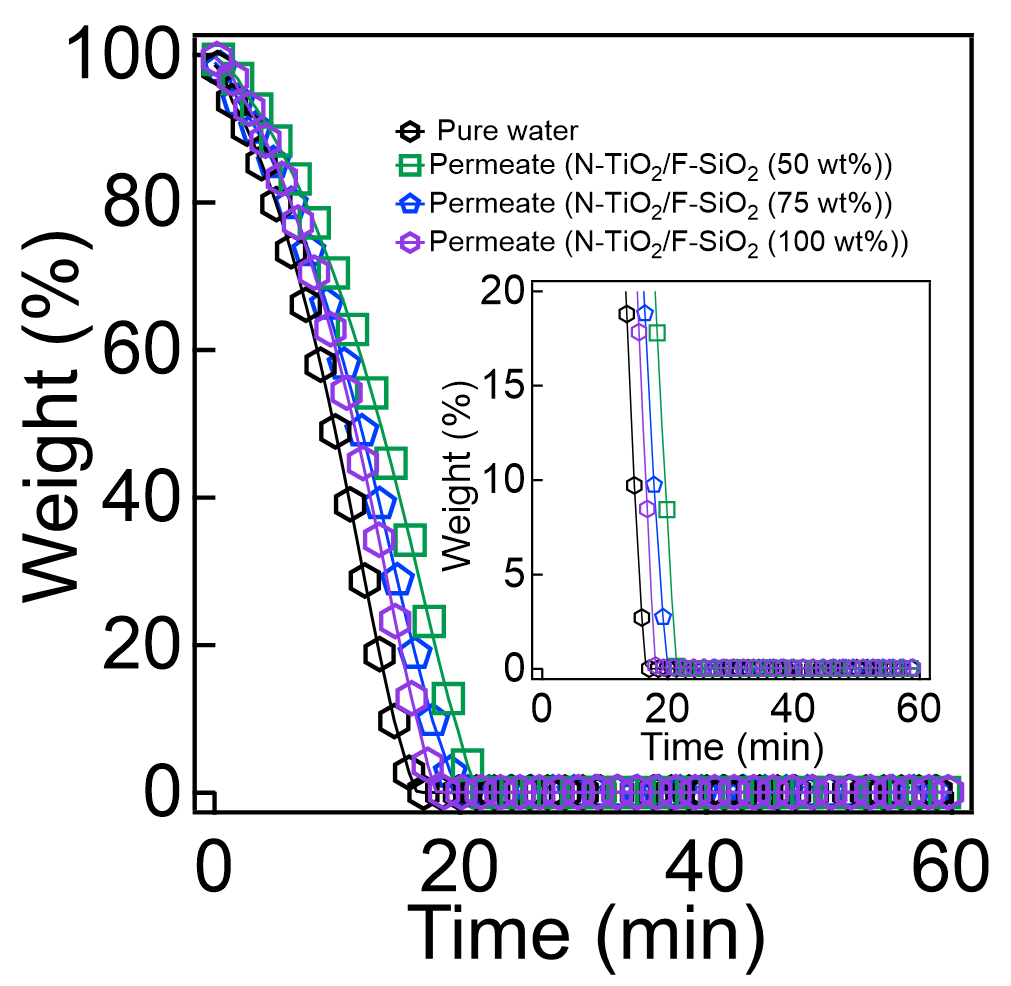


**Figure S4.** TGA data of the water-rich permeates after the separation of SDS-stabilized n-hexadecane-in-water emulsion by utilizing the mesh coated with various compositions of N-TiO_2_/F-SiO_2_.

**Section 5. Calculating the values of *r*_m_, *R*_c_, *f*_c(T)_(*t*_i_ = 0), and *f*_c(F)_(*t*_i_ = 0)**

To predict the values of water-rich permeate flux using equation (4) in the main text, we need to determine the values of the resistance per unit area of the mesh to the permeation of water-rich permeate caused by mesh itself (*r*_m_), and that caused by oil contamination (*R*_c_), as well as the initial area fraction of the oil-contaminated regions for N-TiO_2_ and F-SiO_2_ (*f*_c(T)_(*t*_i_ = 0) and *f*_c(F)_(*t*_i_ = 0)) at the start of visible light illumination.

(i) Calculating *r*_m_ values: A mesh was first subjected to DI water for 60 minutes at Δ*P* = 13.0 kPa ± 0.7 kPa by using the cross-flow apparatus. The *J*(*t*=60 minutes) values were measured. Given that the *r*_m_ is expressed by:

$r_{m}=\frac{\Delta P}{\left( J\left( t \right) \right)\mu}$ (S1)

The *r*_m_ values can be obtained by plugging in Δ*P* = 13.0 kPa and *J*(*t* = 60 minutes). Note that the values of *R*_c_ and *f*_c_ (*t* = 60 minutes) are zero because DI water does not cause any contamination. **Table S1** lists the *r*_m_ values for the mesh coated with N-TiO_2_/F-SiO_2_ with varied compositions.

| **N-TiO_2_/F-SiO_2_ composition** | ***r*_m_ (L^-1^ m^2^ )** |
| --- | --- |
| N-TiO_2_/F-SiO_2_ (50 wt%) | 43,333 ± 500 |
| N-TiO_2_/F-SiO_2_ (75 wt%) | 38,172 ± 500 |
| N-TiO_2_/F-SiO_2_ (100 wt%) | 29,917 ± 300 |

**Table S1.** The calculated *r*_m_ values for the mesh coated with N-TiO_2_/F-SiO_2_ with varied compositions.

(ii) *R*_c_ values: A mesh was subjected to n-hexadecane-in-water emulsion (1:9 volumetric ratio, n-hexadecane:water) that was stabilized by sodium dodecyl sulfate (SDS) for 60 minutes at Δ*P* = 13.0 kPa ± 0.7 kPa using a cross-flow apparatus. By rearranging equation (3) in main text, the *R*_c_ is given as:

$R_{c} =A\left( \frac{\Delta P}{\left( J\left( t_{i} \right) \right)\mu}-r_{m} \right)\left( 1-\left( \begin{aligned} \\ f_{\left( F \right)}\left[ \frac{k_{a\left( T \right)}}{K_{\left( T \right)}}-\left( \frac{k_{a\left( T \right)}}{K_{\left( T \right)}}-f_{c\left( T \right)}\left( t_{i}=0 \right) \right)e^{-\left( K_{\left( T \right)} \right)t_{i}} \right]\times f_{\left( T \right)} \\ \\ +\left[ \frac{k_{a(F)}}{K_{\left( F \right)}}-\left( \frac{k_{a(F)}}{K_{\left( F \right)}}-f_{c(F)}\left( t_{i}=0 \right) \right)e^{-\left( K_{\left( F \right)} \right)t_{i}} \right]\times f_{(F)} \end{aligned} \right) \right)$ (S2)

We obtained the *R*_c_ values by plugging in the variables that were used in the *r*_m_ calculation (i.e., Δ*P* = 13.0 kPa, *A*= 42 cm^2^) as well as the *r*_m_ values reported in **Table S1**. The calculated *R*_c_ values for the mesh coated with N-TiO_2_/F-SiO_2_ with varied compositions are summarized in **Table S2**.

| **N-TiO_2_/F-SiO_2_ composition** | ***R*_c_ (L^-1^ m^4^)** |
| --- | --- |
| N-TiO_2_/F-SiO_2_ (50 wt%) | 16.9 ± 0.6 |
| N-TiO_2_/F-SiO_2_ (75 wt%) | 20.4 ± 0.5 |
| N-TiO_2_/F-SiO_2_ (100 wt%) | 23.4 ± 0.6 |

**Table S2.** The calculated *R*_c_ values of the mesh coated with varied compositions of N-TiO_2_/F-SiO_2_.

(iii) *f*_c(T)_(*t*_i_ = 0) and *f*_c(F)_(*t*_i_ = 0) values: We calculated the values of *f*_c(T)_ and *f*_c(F)_ at the onset of visible light illumination (*t*_i_ = 0) by plugging the values of rate constants (i.e., *k*_a_, *k*_d_, and *k*_p_) and *θ**_w,o_ in equation (5) in the main text. **Table S3** lists the calculated values of *f*_c(T)_(*t*_i_ = 0) and *f*_c(F)_(*t*_i_ = 0), as well as the measured values of *θ**_w,o_.

| **N-TiO_2_/F-SiO_2_ composition** | *θ**_w,o_ | *f*_c(T)_(*t*_i_ = 0) | *f*_c(F)_(*t*_i_ = 0) |
| --- | --- | --- | --- |
| N-TiO_2_/F-SiO_2_ (50 wt%) | 89°±3° | 0.80 | 0.1 |
| N-TiO_2_/F-SiO_2_ (75 wt%) | 46°±1° | 0.80 | 0.1 |
| N-TiO_2_/F-SiO_2_ (100 wt%) | 63°±3° | 0.80 | - |

**Table S3.** The calculated values of *f*_c(T)_ and *f*_c(F)_, as well as the measured values of *θ**_w,o_ for the mesh coated with N-TiO_2_/F-SiO_2_ with varied compositions at the onset of visible light illumination (*t*_i_ = 0).

**Section 6. Effect of visible light intensity on the *k*_p_ values**

We determined the *k*_p_ values for N-TiO_2_ surface upon being illuminated by visible light with varied intensities (*I* = 30 and 100 mW cm^-2^) by fitting equation (5) in the main text to the cosine values of the experimentally measured *θ^*^*_w,o_ values (**Figure S5**). The *k*_p_ values were obtained as 3.5 × 10^-4^ s^-1^ and 8.9 × 10^-3^ s^-1^ for *I* = 30 and 100 mW cm^-2^, respectively, which are lower in comparison to the *k*_p_ value (*k*_p_ = 9.8×10^−3^ s^−1^) under visible light illumination with intensity of *I* = 198 mW cm^-2^. Note that the highest intensity using our visible light source is *I* = 198 mW cm^-2^.


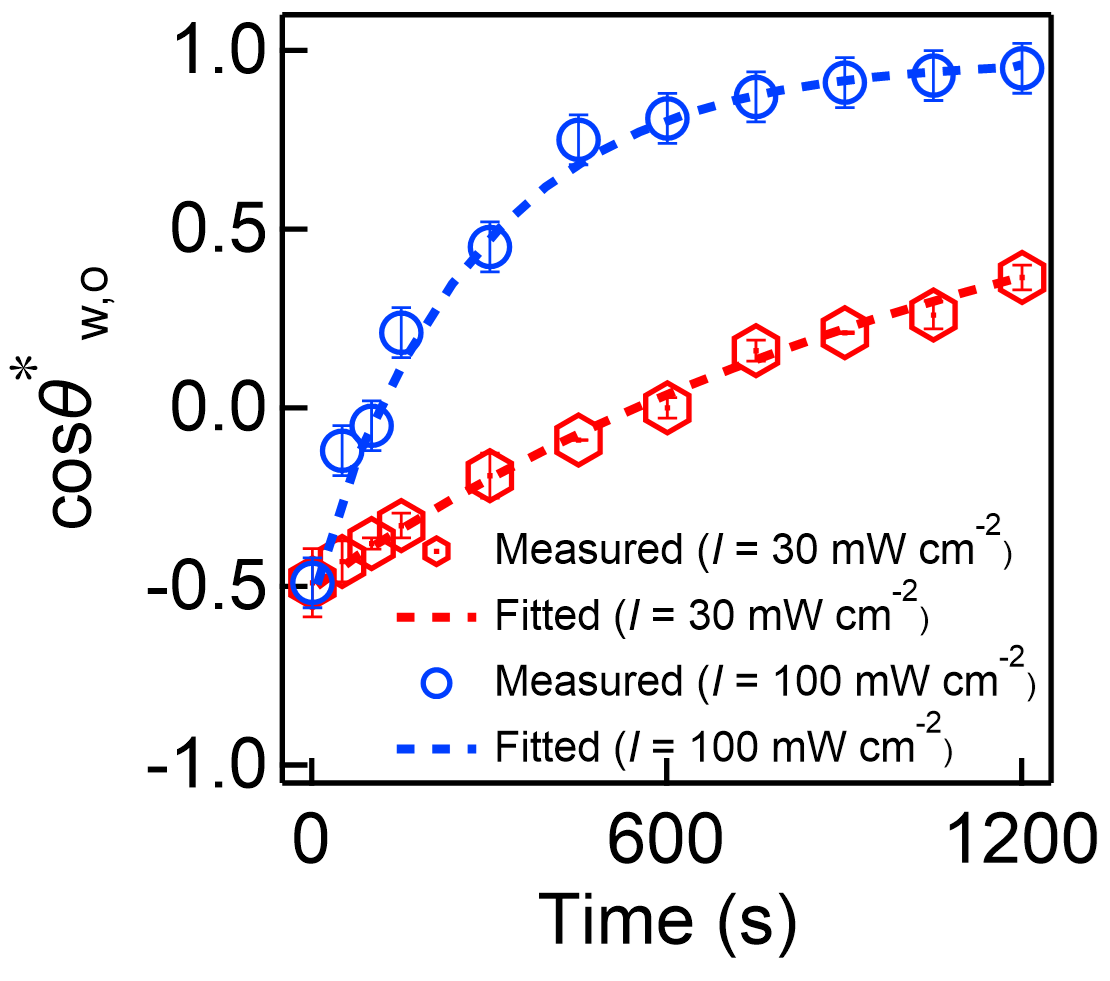


**Figure S5.** Plot of the cosine values of the measured apparent water contact angle (*θ^*^*_w,o_) on N-TiO_2_ surface submerged in oil as a function of visible light illumination (*I* = 30 and 100 mW cm^-2^) time. The *k*_p_ value for oil on N-TiO_2_ was determined by fitting the relation (equation (5) in the main text).

**References**

1 ASTM, A. D3359-17 Standard Test Methods for Rating Adhesion by Tape Test. *West Conshohocken, PA: ASTM International* (2017).

2 Camin, D. L., Forziati, A. F. & Rossini, F. D. Physical properties of n-hexadecane, n-decylcyclopentane, n-decylcyclohexane, 1-hexadecene and n-decylbenzene. *The Journal of Physical Chemistry* **58**, 440-442 (1954).
